# Author's reply

**DOI:** 10.4103/0974-7788.64402

**Published:** 2010

**Authors:** Kishor Patwardhan

**Affiliations:** *Department of Kriya Sharir, Faculty of Ayurveda, IMS, BHU, Varanasi 221 005, Varanasi, Uttar Pradesh, India E-mail: patwardhan.kishor@gmail.com*

Sir,

In response to the communication by Dr. Sanjeev Rastogi we are willing to give our following comments:

1. Apart from the challenges of Ayurveda education that we have recognized in the present study,[1] we have also observed that the Ayurveda graduates are not trained adequately in basic clinical skills[2] and this is probably the major cause of diffident clinicians being produced. Unless the memory-oriented and theory-oriented teaching does not transform into clinically oriented practical training, the problem is probably going to remain.
2. There is an urgent need of establishing a national level body for taking care of the following.
  - Educational research is carried out in Ayurveda and suitable recommendations are put forth from time to time to ensure the relevance of Ayurveda education.
  - Strict regulatory norms are implemented while granting approvals to the institutions.
  - Uniform pay packages and regular promotions are ensured to attract and retain good teachers and clinicians in the education system.
  - NET-like compulsory national level screening test is introduced to assess the quality of the aspiring teachers before declaring them eligible for lectureship.
3. At present, India follows the policy model of "parallel approach", where traditional systems of medicine and Allopathy are segregated.[3] Implementation of the policy model of "integrated approach", where all streams of medicine are integrated at all the levels of education and practice, as being followed in China and Vietnam,[4] may be the eventual solution for this problem.
